# Monthly Summary

**Published:** 1888-01

**Authors:** 


### Monthly Summary.

---

Two Cases of Epulis.—*By Mr. C. Rippon, L. D. S. I.* —During the past six months I have had in my own practice two distinct cases of epulis, one occurring in the inferior and one in the superior maxilla. First:—Miss W——, a domestic servant, about 24 years of age, applied to me respecting the difficulty she experienced in closing her mouth properly, and particularly in masticating her food. On examining her mouth, I found that all her back teeth, upper and lower, were decayed down to the level of the gum. so that when her mouth was closed the lower incisors were pressing upon the roof of the mouth and the gums touching each other behind. Between the second bicuspid and the first molar roots (lower left side) there was a small epulis about the size of a pea, just sufficiently large enough to prevent the jaws from shutting properly; consequently during mastication, this growth frequently got squeezed and so caused her annoyance, though not very painful. In figure, the tumour was nearly round and attached by a narrow neck to the gum between the above-mentioned teeth. On the lingual aspect it very much resembled true gum, but of a bluish red colour, probably caused by the frequent squeezing between the jaws. I extracted both bicuspid and molar roots, and while removing the latter the growth and alveolus to which it was attached came away with it. I have since extracted the whole of the roots both upper and lower, and replaced them with artificial dentures, which she is now wearing with comfort, and there is no sign of a recurrence of the tumor.

Case 2.—Miss H——, age 22. In this case the patient had noticed the growth for the past ten or eleven years, and had attained a considerable size (about the size of a filbert nut); it was entirely free from pain, but disfigured the patient very much, as it was partially exposed, the lip not being able to cover it. The tumor occupied the position between the right superior lateral and canine on the labial aspect, and in shape very much resembled fig, 81, page 161, "Salter's Dental Pathology and Surgery." The teeth were very irregular, at the same time very much decayed. The growth had all the appearances of healthy gum tissue made up into a lobular mass, and attached by a broad base extending from the mesial side of the lateral to the centre of the canine. I extracted both the lateral and canine, extirpated the growth and removed a considerable portion of the adjacent bone, the dividing wall of the socket being very friable. In a fortnight after the operation I took a cast of the mouth, which was apparently healing very rapidly. I have not seen the patient since, but have heard that she is perfectly well again and no signs of a recurrence. The operation proved very successful with cocaine, the patient only complaining of pain when the bone was being cut rather deeply.—*London Dental Record.*

---

LODGMENT OF A TOOTH-PLATE IN THE GULLET FOR FIFTEEN MONTHS.—*By Henry E. Bridgman, L. R. C. P. Lon.*—At 2 a. m. on April 17th, 1886, Thomas H——, aged twenty eight, was brought to my surgery. He informed me that he had gone to bed, as was his habit, wearing a small plate, to which were attached four false teeth. He awoke feeling the plate slipping in his throat, and he could, he said, still feel it at the top of his gullet. The patient was nervous and excited· he frequently retched, and expectorated blood-stained saliva; dyspnœa and dysphagia were marked. I examined the pharynx carefully with the finger, but failed to feel any foreign body. With a pair of throat forceps I seized something that I believed to be the plate, but on making an attempt to withdraw it, the dyspnœa increased, the patient struggied, and the forceps slipped. After several fruitless attempts to seize the plate a second time, I desisted, and sent my patient to the Burton-on-Trent Infirmary. There probangs were passed into the stomach without meeting with any obstruction. The patient was watched for a few days, and then discharged, the dyspnœa and dysphagia having disappeared.

I saw the man a few weeks afterwards, when he was again suffering frem dyspnœa, had a hard, frequent cough, and was expectorating copiously a tenacious mucus, tinged with blood. I continued to see him from time to time until Christmas, 1886. On July 25th, 1887, he presented himself again at my surgery, bringing with him the plate with false teeth attached, saying that he had continued much in the same condition as when I had last seen him, being frequently unable to go to his work, on account of cough and difficult breathing, uutil July 21st, when, being rather worse than usual, he felt, after a violent fit of retching, something in the back of his throat. when, by means of his thumb-nail, he hooked out the false teeth, which he brought with him and which he had lost just fifteen months since.—*The Lancet.*

---

A CASE of somewhat remarkable character, says the *Lancet*, is at the present time in the London Temperance Hospital, under the care of Dr. R. J Lee. A girl fifteen years of age had the last molar tooth in the lower jaw on the right side removed about six weeks ago. No anæsthetic was administered. She was in perfect health at the time. Half an hour after the operation she began to yawn, and has continued to do so constantly since. One yawn succeeds another without interruption, and with an interval of two or three seconds. Galvanism had been tried without effect, and other remedies previous to admission into the hospital. Three days afterwards the yawning changed to sneezing, and recently she has suffered from constant and rapidly succeeding fits of sneezing each of which paroxysms appears to begin with a yawn. She seems to have no power of controlling herself, or only to a very slight extent; and if she sttempts to do so, the next sneeze is more violent.

---

TREATMENT OF DIARRHŒA BY IODOFORM AND CHARCOAL.—Picchini treated eight cases of diarrhœa, with symptoms of fermentations, with the following:

R Iodoform, .................................grs. 9.

Ether, .......................................... ℥ $3\frac{1}{8}$.

Vegetable charcoal, finely powered,... ℥ $3\frac{1}{8}$.

Glycerine, ad .................................. ℥ 12.

The iodoform must be dissolved in the ether, and the powdered charcoal thoroughly mixed. After the ether has evaporated, the glycerine should be added. To take in twenty-four hours, by teaspoonful or tablespoonful, suspended in a glass of water.—*Journal de Medicine.*

